# Supplementary material for: Patterns of Intron Gain and Loss in Fungi
Source: PLoS Biol. 2004 Nov 30;2(12):e422. doi: 10.1371/journal.pbio.0020422 (PMC532390; doi:10.1371/journal.pbio.0020422)
Supplement: Table S1 — Also available at http://genes.mit.edu/NielsenEtAl/. (4.3 MB ZIP). [file pbio.0020422.st001.zip › NielsenEtAl/html/625.html]

AN1446.1.NCU09230.1.MG10380.1.FG01177.1


```
 CLUSTAL W (1.82) Multiple Sequence Alignments - Introns Inserted


Sequence 1: NCU09230.1	417 aa
Sequence 2: FG01177.1	423 aa
Sequence 3: AN1446.1	410 aa
Sequence 4: MG10380.1	426 aa
Alignment Length: 431 aa
Number Identitical Residues: 249 aa
Alignment Score (without introns) 11021


MG10380.1 	MTASNLHEQAPAVAAVAHQQQQQQQQSSRGFATRAVHAGSSPDPTTGAVIPA~ISLSTTF
NCU09230.1	MTAS--SDHPITTPPRAPSPVHG-------FGTLAVHAGSPHDPATGAVIEA~ISLSTTF
FG01177.1 	MSSAPLNSDPVATPPRPPSPVHN-------FGTLAVHAGAPHDPATGAVIES~ISLSTTF
AN1446.1  	MTMMAGHPDLPSNGQNGDSNTHQQRQ----FATLAVHAGAPHDPTTGAVIAP0ES-----
          	*:      .  :      .  : . .    *.* *****:. **:***** .  *     

MG10380.1 	RQSAPSQPVGLYEYSRSANPNR~DNFEEAVASLENARHGFAFSSGSATTAVVLQSLASGS
NCU09230.1	AQTAVGKPVGEFEYSRSSNPNR~ANFEKMVAALEHAKYALAYSSGSATTANILQSLAAGS
FG01177.1 	AQSAVGKPVGEYEYSRSSNPNR~TNFETAVAALEHAKYALAFSSGSATTATILQSLAAGS
AN1446.1  	----VGKPVGLYEYTRSSNPNR2DNFEEAVASLEHAKYALAFSSGSATTATILHSLAPGS
          	     .:*** :**:**:****  ***  **:**:*::.:*:******** :*:***.**

MG10380.1 	HVISVSDVYGGTHRYFTQVAQAHNIEVTFSHDIVADVGSQIRP-DTKLIWIESPSNPTLS
NCU09230.1	HVISVSDVYGGTHRYFTQVAKAHGVKVTFTPEIEVDIRDHITD-ATKLVWIETPSNPTLR
FG01177.1 	HVISVSDVYGGTHRYFTQVAKAHGVKVTFTPEIEVDIAEHITP-DTRLIWIETPSNPTLR
AN1446.1  	HVVSVSDVYGGTHRYFTKVAAAHGVNVSFSSCLELDVEKLIRPNETKLVWIETPSNPTLA
          	**:**************:** **.::*:*:  :  *: . *  . *:*:***:****** 

MG10380.1 	LVDIRAVADAAHARGVLVVVDNTFLSPYIQNPLNLGADIVVHSVTKYINGHS~DVVMGVA
NCU09230.1	LVDIRAVATAAHERGILVVVDNTFLSPYVQNPLDHGADIVVHSVTKYINGHS~DVVMGVA
FG01177.1 	LVDIRAVVTEAHKHGVLVVVDNTFLSPYVQNPLDFGADIVVHSVTKYINGHS~DVVMGVA
AN1446.1  	LVDIRKVAAVAHRHGVLVVVDNTFMSPYVQNPLDHGADVVIHSVTKYINGHS0DVLMGVA
          	***** *.  ** :*:********:***:****: ***:*:*********** **:****

MG10380.1 	ACNDDALASRLRFLQNAIGAVPSAFDSWLAHRGLKTLHLRAREASRNAAAVAAFLETSPY
NCU09230.1	AFNSDELYARLSFLQNAIGAVPSAFDSWLAHRGAKTLHLRAREATTNATAIAHALEASPL
FG01177.1 	AFNSDELKARLSFLQNAIGAVPSAFDSWLAHRGLKTLHLRAREASRNADAVARALEASPL
AN1446.1  	AFNSDELKERFTFLQNAIGAVPSPFDCWLAHRGLKTLHLRAREATANATAVALALESSPH
          	* *.* *  *: ***********.**.****** **********: ** *:*  **:** 

MG10380.1 	VISVNYPGLESHPQRDIARRQHRDGMGGGMLSFRVKGGLVAADSFCSTTRVFTLAESLGG
NCU09230.1	VISVNYPGLESHPHRAIALKQHRNGMGGGMLSFRIHGGHAAAEKFCQYTKIFTLAESLGG
FG01177.1 	VIAVNYPGLDSHPHRHIAKKQHRDALGGGMLSFRIQGGHAAAERFCQVTKIFTLAESLGG
AN1446.1  	VISVNYPGLNSHPNREIAVKQHRKGMGGGMLSFRIKGGHKAAHLFCEYTKIFTLAESLGG
          	**:******:***:* ** :***..:********::**  **. **. *::*********

MG10380.1 	VESLVELPSVMTHAGIPRQSREAVGIYDDLIRISCGIEEAEDLVADVRQALEAAALASKL
NCU09230.1	VESLCEIPSSMTHAGIPKAQREAVGIFDDLVRISCGVEDAEDLKADVLQALERAVADA-A
FG01177.1 	VESLVEVPSSMTHAGIPRDQREAVGIFDDLVRISCGVEDAQDLTNDVLQALEKATSAAKA
AN1446.1  	VESLCEVPSSMTHAGIPKEEREAAGVYDDLVRMSCGIEDVEDLTADTMQALERAVAAS--
          	**** *:** *******: .***.*::***:*:***:*:.:**  *. **** *.  :  

MG10380.1 	HSSS---GNGNGH-
NCU09230.1	NGVS-NGVNGTH--
FG01177.1 	NGLNGNGVNGSNGH
AN1446.1  	QALE----NGSA--
          	:. .    **.
```
